# Mass Spectrometric and Spectrofluorometric Studies of the Interaction of Aristolochic Acids with Proteins

**DOI:** 10.1038/srep15192

**Published:** 2015-10-16

**Authors:** Weiwei Li, Qin Hu, Wan Chan

**Affiliations:** 1 Department of Chemistry, The Hong Kong University of Science and Technology, Clear Water Bay, Kowloon, Hong Kong

## Abstract

Aristolochic acid (AA) is a potent carcinogen and nephrotoxin and is associated with the development of “Chinese herb nephropathy” and Balkan endemic nephropathy. Despite decades of research, the specific mechanism of the observed nephrotoxicity has remained elusive and the potential effects on proteins due to the observed toxicity of AA are not well-understood. To better understand the pharmacotoxicological features of AA, we investigated the non-covalent interactions of AA with proteins. The protein-binding properties of AA with bovine serum albumin (BSA) and lysozyme were characterized using spectrofluorometric and mass spectrometric (MS) techniques. Moreover, the protein-AA complexes were clearly identified by high-resolution MS analyses. To the best of our knowledge, this is the first direct evidence of non-covalently bound protein-AA complexes. An analysis of the spectrofluorometric data by a modified Stern−Volmer plot model also revealed that both aristolochic acid I (AAI) and aristolochic acid II (AAII) were bound to BSA and lysozyme in 1:1 stoichiometries. A significantly stronger protein binding property was observed in AAII than in AAI as evidenced by the spectrofluorometric and MS analyses, which may explain the observed higher mutagenicity of AAII.

*Aristolochia*-derived aristolochic acid (AA) is one of the most potent carcinogens and is listed as a Class I human carcinogen, with aristolochic acid I (AAI) and aristolochic acid II (AAII) being the major components^1^,^2^. Extensive studies have demonstrated that AA forms covalently bonded DNA adducts upon metabolic activation, and these DNA–AA adducts have been identified in multiple internal organs of AA-dosed rats^3^,^4^,^5^. Moreover, AA can induce cancers in laboratory rodents, with a high frequency of AT → TA transversions in tumor tissues^6^,^7^.

There is significant evidence in the literature that suggests an association between the continuous intake of AA-containing herbal remedies with the development of “Chinese herb nephropathy” in Belgian women participating in a slimming regimen^8^,^9^. Emerging evidence also suggests that chronic food poisoning by AA-contaminated wheat flour is a leading cause of Balkan endemic nephropathy, a peculiar kidney disease that is prevalent among farmers living along the Danube River^7^,^10^,^11^. Although AA has been traditionally considered to be strongly nephrotoxic, the molecular mechanism underlying the kidney fibrotic process remains unclear^9^,^11^.

The interactions of drugs and toxins with proteins and enzymes are characterized by their respective therapeutic and toxicological effects^12^,^13^,^14^,^15^. For instance, organophosphate flame retardants, such as triphenyl phosphate, disrupt the conformational structure of the DNA-binding domain in the p53 tumor suppressor protein, affecting its role in cancer prevention^16^. However, the interactions between proteins and AA are not well understood and investigations into their relationship are relatively new^17^. In addition, interactions of proteins with AAII have not yet been examined in previous studies^17^.

In this study, we report the protein binding properties of AA using spectrofluorometric and mass spectrometric (MS) methods. Using high-resolution mass spectrometry (HR-MS) coupled with electrospray ionization (ESI), we present the first direct evidence of non-covalent interactions between AA and proteins. The protein-binding constant and the binding stoichiometries of AAI and AAII were also determined using the fluorescence-quenching property of AA.

## Results and Discussion

### Spectrofluorometric analysis of the protein-AA complexes

**Figure 1:**
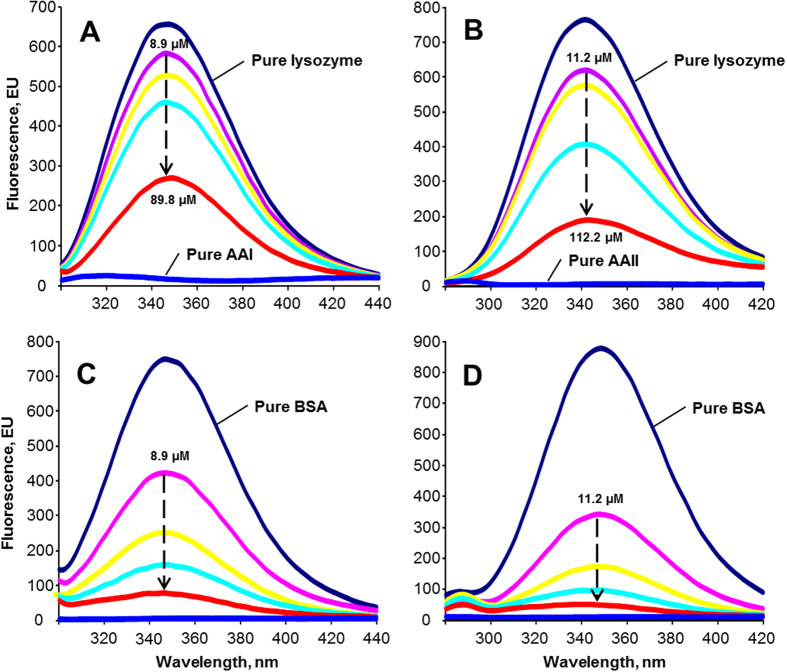
The effects of (**A**) AAI and (**B**) AAII on quenching the intensity of the native fluorescence of lysozyme, together with efficiency of (**C**) AAI and (**D**) AAII in quenching the intrinsic fluorescence of BSA under physiological conditions (pH 6.9 at 298 K). The concentrations of both lysozyme and BSA were 11.0 μM. The concentrations of AAI and AAII ranged from 8.9 to 89.8 μM and from 11.2 to 112.2 μM, respectively.

Although AA is a rigid polycyclic aromatic hydrocarbon, no molecular fluorescence was observed (Fig. 1)^18^. However, intrinsic fluorescence was observed for proteins that contained aromatic amino acids (i.e., tryptophan, tyrosine, and phenylalanine)^14^,^19^. Similar to many other drugs^12^,^19^,^20^,^21^, AA is able to quench the native fluorescence of human serum albumin (HSA)^17^.

Using the fluorescence quenching properties of AA, we investigated the protein-binding properties of AA on two of the most common proteins, BSA and lysozyme. As shown in Fig. 1A, AAI induced a dosage-dependent quenching of the native fluorescence of lysozyme. A similar, but stronger fluorescence quenching effect was observed for AAII (Fig. 1B), indicating that AAII had a stronger interaction with lysozyme than AAI.

The spectrofluorometric analyses also revealed that AA exhibited a stronger quenching effect on the native fluorescence of BSA than on lysozyme (Fig. 1). As was observed for lysozyme, AAII (Fig. 1D) was found to be more efficient than AAI in quenching the native fluorescence of BSA (Fig. 1C). These results suggest that AAII had a higher protein binding affinity and was a more efficient fluorescence quencher than AAI. This phenomenon may be attributed to the smaller molecular size of AAII relative to AAI, which reduces the probability of steric hindrance within protein binding sites.

**Figure 2:**
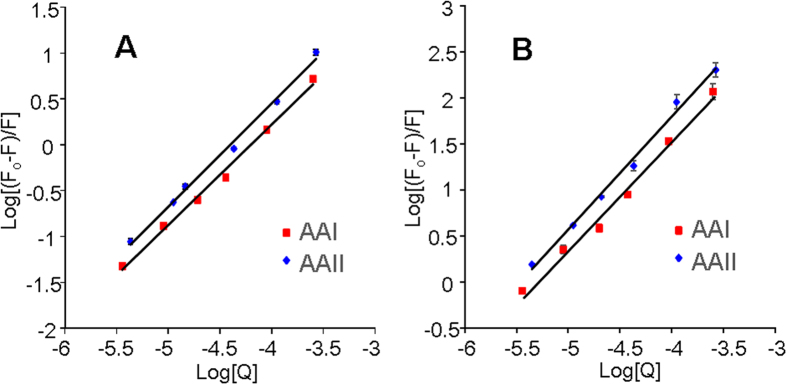
Plots of log(F_0_ − F)/F versus log[Q] for binding constants and binding stoichiometries of AAI and AAII with (**A**) lysozyme and (**B**) BSA.

Using equation 1,^26^, the binding constant (*K*) and the stoichiometry (*n*) of AA binding to BSA and lysozyme were determined. Figure 2A,B show the modified Stern−Volmer plot for lysozyme and BSA, respectively. *K* and *n* are the intercept and the slope of the plots, respectively. The binding constants and stoichiometries of AA binding to lysozyme and BSA under physiological conditions (pH 6.9, 298 K) are summarized in Table 1.

Using equation 1, the analyses of the spectrofluorometric data of BSA and lysozyme revealed similar binding stoichiometries (*n* = 1) for both proteins, indicating that for every AA-associated protein molecule, one molecule of AA was bound. A similar 1:1 binding stoichiometry was also observed in a previous study investigating the interaction of AAI with HAS^17^.

However, the binding constant of AAI observed in this study (1.89 × 10^6^) was much larger than that observed by Wu *et al*. (4.1 × 10^5^)^17^. This discrepancy may be due to differences in the chemical composition of BSA and HSA. HSA has only one indole-containing tryptophan residue, whereas BSA contains two tryptophan residues^17^, which creates stronger molecular interactions with AA. Thus, a larger binding constant was observed for BSA.

### Mass spectrometry analysis of the protein-AA complexes

**Figure 3:**
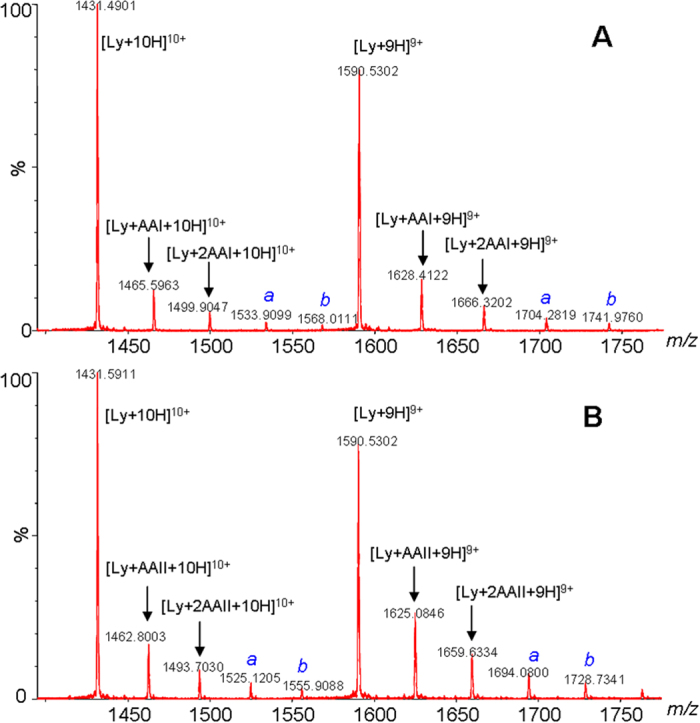
ESI-MS analyses of mixtures from mixing 11.0 μM lysozyme solutions with (**A**) AAI and (**B**) AAII at 1:10 molar ratios. *a* and *b* designate lysozyme-AA complexes with binding stoichiometries of 1:3 and 1:4, respectively.

ESI-MS is a powerful tool for investigating the non-covalent interactions of macrobiomolecules with toxins and was used in this study to characterize the non-covalent complexes between AA and lysozyme^15^,^22^,^23^. The HR-MS analyses of lysozyme mixed with 10 equivalents of AA revealed the clear formation of lysozyme-AA complexes (Fig. 3). To the best of our knowledge, this is the first direct evidence demonstrating the existence of protein-AA complexes.

**Figure 4:**
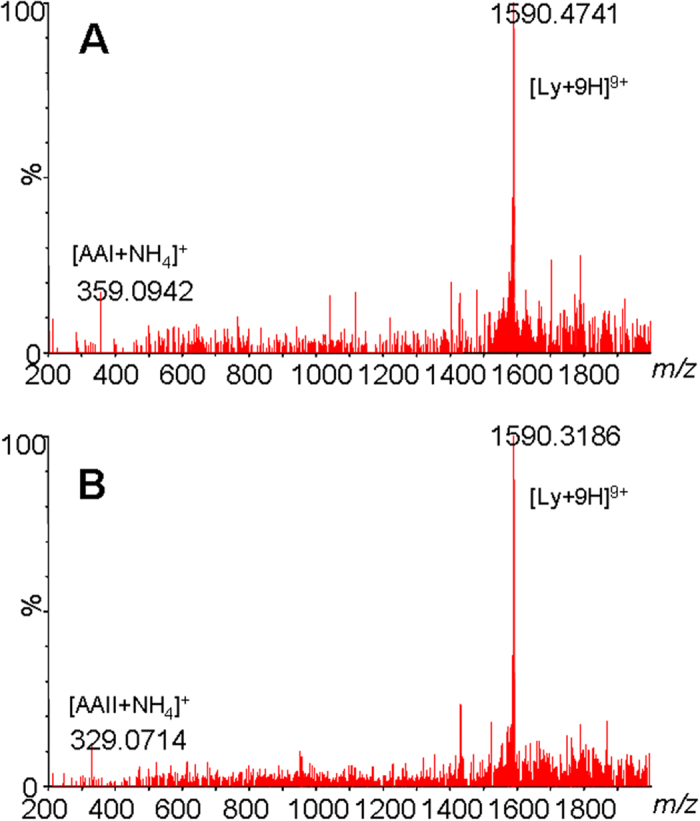
MS/MS analyses of the [M + 9H]^9 + ^ions of the lysozyme-AAI (**A**, *m/z* 1628) and lysozyme-AAII (**B**, *m/z* 1625) complexes.

High-accuracy MS analyses of the complexes revealed a close correlation between the measured and theoretical *m/z* values, with the mass errors less than 50 ppm. MS/MS analyses of the lysozyme-AA complexes (Fig. 4) showed characteristic fragment ions of the unmodified protein and the corresponding AA. These results demonstrated that lysozyme and AA were linked together by weak non-covalent interactions and the complexes were therefore easily dissociated upon collision-induced dissociation MS/MS analyses.

Results from the ESI-MS analysis show the clear formation of lysozyme-AA complexes with binding stoichiometries of 1:1, 1:2, 1:3, and 1:4 for both AAI (Fig. 3A) and AAII (Fig. 3B). These binding stoichiometries were inconsistent with the 1:1 stoichiometry predicted using equation 1 and with the results from the spectrofluorometric analysis^17^. Whereas the 1:1 binding stoichiometry is believed to have resulted from the specific binding of AA to the hydrophobic cavities of subdomain IIA of serum albumin^17^, the 1:2, 1:3, and 1:4 binding stoichiometries could have resulted from the nonspecific binding of AA to lysozyme at high concentrations of AA^15^.

**Figure 5:**
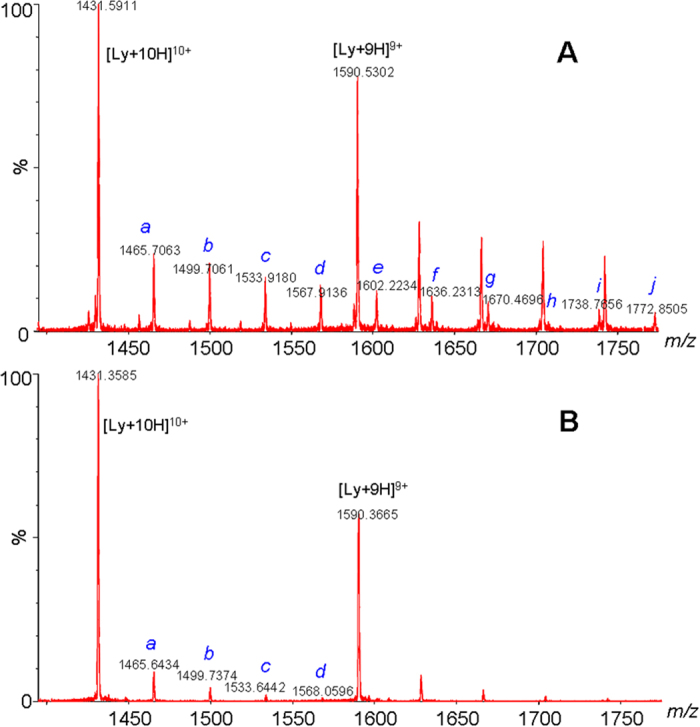
ESI-MS analyses of mixtures of 11.0 μM lysozyme solutions with AAI in molar ratios of (**A**) 1:100 and (**B**) 1:3. Letters *a* to *j* designate lysozyme-AAI complexes with binding stoichiometries from 1:1 to 1:10.

This hypothesis was validated by an ESI-MS analysis of a mixture of lysozyme and AAI at a molar ratio of 1:100. We observed the more extensive formation of lysozyme-AA complexes with binding stoichiometries ranging from 1:1 to 1:10 (Fig. 5A). As shown in Fig. 3 and Fig. 5, there was a dose-dependent formation of the protein-AA complexes in the HR-MS analyses of lysozyme solutions mixed with AAI at molar ratios of 1:3 (Fig. 5B), 1:10 (Fig. 3A), and 1:100 (Fig. 5A).

**Figure 6:**
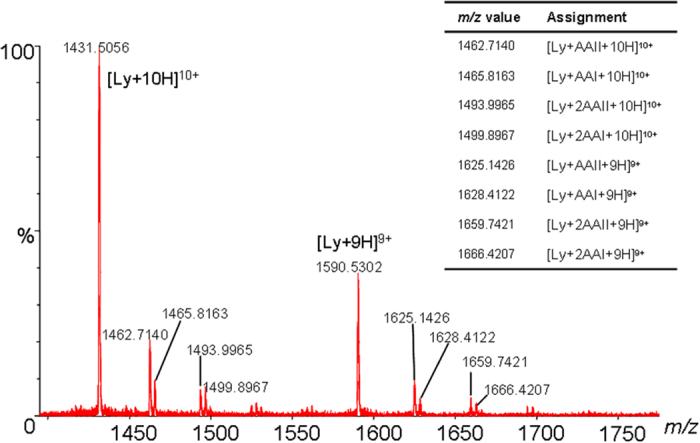
ESI-MS analyses of 11.0 μM lysozyme solutions with a mixture of AAI and AAII (1:1) at a 1:10 molar ratio.

The spectrofluorometric analysis indicated a larger binding constant with lysozyme for AAII than AAI. Similarly, the HR-MS analysis of lysozyme mixed with AAII also showed significantly higher levels of lysozyme-AAII complexes than lysozyme-AAI complexes. The stronger binding of AAII to lysozyme reduced its dissociation during the ionization process and resulted in a higher concentration of the lysozyme-AAII complex than the lysozyme-AAI complex in the HR-MS analysis. A similar phenomenon was also observed in a competition study that analyzed a lysozyme solution mixed with a mixture of AAI and AAII (1:1). Significantly higher levels of AAII complexes than AAI complexes were observed (Fig. 6).

Our results showed that the protein binding constant of AAII was higher than that of AAI, which is consistent to a previous study demonstrating higher levels of AAII than AAI in plasma sample from AA-treated rats^24^. The higher binding affinity to the drug-carrying serum albumin protein would result in a higher proportion of AAII in blood circulation, thereby reducing AAII elimination from the body. Thus, a higher plasma concentration of AAII was observed in AA-treated rats^24^.

The high binding affinity of AAII to proteins may also explain its observed higher mutagenicity^5^,^24^,^25^ and nephrotoxicity than that of AAI. Being able to bind stronger to the drug-carrying serum albumins, a larger proportion of AAII than AAI was in its protein bound form. Therefore, AAII will be stored better and longer than AAI in the body as protein bound complex. As the unbounded AAII is being metabolized and eliminated, AAII will continuously being released from its protein complex to exhibit its toxicological effects.

## Conclusions

Using the fluorescence-quenching properties of AA, we compared the protein binding properties of AAI and AAII. For the first time, our study revealed that AAII has greater affinity for serum albumin proteins and enzymes than AAI, which may indicate the higher mutagenicity of AAII. Moreover, results from HR-MS analyses provide direct evidence for the formation of protein complexes with AAI and AAII. We believe that the results from these studies will enhance our understanding of the relative nephrotoxicity and carcinogenicity of AAI and AAII.

## Methods

### Caution

AA is a potent human carcinogen and nephrotoxin and should be handled with care.

### Chemicals

All chemicals and reagents were of the highest purity available and were used without further purification unless otherwise noted. A mixture of AA consisting of AAI and AAII (1:1) was purchased from Acros (Morris Plains, NJ). AAI, bovine serum albumin (BSA), and egg white lysozyme were obtained from Sigma (St. Louis, MO). AAII was isolated from *Aristolochiae Cinnabarina* using previously reported methods^18^ and purified by HPLC (Supplementary Fig. S1). HPLC-grade methanol was obtained from Tedia (Fairfield, OH). Deionized water was further purified with a Milli-Q ultrapure water purification system (Billerica, MA) and used throughout the study.

### Instrumentation

HR-MS analyses were performed on a Waters Xevo G2 Q-TOF mass spectrometer with a standard ESI interface (Milford, MA). The capillary and cone voltages for the positive ESI-MS analysis were set at 2.5 kV and 12 V, respectively. The TOF MS was calibrated using 0.5 mM sodium formate solution before analyses. During the analysis, a lock-mass solution of Leucine Enkephalin solution (2 ng/μL^−1^) was continuously being infused into the MS via the lock-spray interface for auto-mass correction. Fluorescence measurements were recorded on a LS55 luminescence spectrometer (PerkinElmer Instruments, UK) at an excitation wavelength of 280 nm for emission wavelengths of 300-450 nm. The HPLC purification of AAII was performed on an Agilent 1100 HPLC (Palo Alto, CA) with a C18 phase column (Shimadzu, Japan).

### Sample preparation

BSA and lysozyme solutions (11.0 μM) were separately prepared by dissolving purified BSA or lysozyme in ammonium acetate buffer (30 mM, pH 6.9) and stored at 4 °C until use. Prior to analysis, 20 μL of AA, AAI or AAII (0, 0.015, 0.04, 0.07, 0.15, 0.4, and 0.9 mg/mL) were added to 200 μL of protein solution and allowed to react for 30 min at ambient temperature. The solution mixtures were then transferred to a quartz cuvette for fluorescence analysis or infused directly into a Q-TOF for HR-MS analysis.

### Statistical analysis

The fluorescence intensity (λ_em_ = 345 nm) of BSA and lysozyme in the presence of AA at different concentrations was processed to obtain the various binding parameters of AA. A modified Stern−Volmer equation^26^,


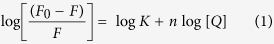


was used in this study to determine the binding constant (*K*) and the binding stoichiometries (*n*) of AA with the representative proteins at physiological conditions.

The protein binding properties of AA were determined by plotting log[(F_0_ – F)/F] against log[Q], where F_0_ and F represent the native fluorescence intensities of the protein in the absence and presence of AA, respectively, and [Q] represents the concentration of the fluorescence quencher.

## Additional Information

**How to cite this article**: Li, W. *et al*. Mass Spectrometric and Spectrofluorometric Studies of the Interaction of Aristolochic Acids with Proteins. *Sci. Rep*. **5**, 15192; doi: 10.1038/srep15192 (2015).

## Supplementary Material

Supplementary Information

## Figures and Tables

**Table 1:** Binding constants and binding stoichiometries of AAI and AAII to lysozyme and BSA under physiological conditions.

		*K*, L/mole	*n*	R^2^	*SD*
Lysozyme	AAI	3.92 × 10^4^	1.09	0.9938	0.0028
	AAII	9.24 × 10^4^	1.13	0.9944	0.0027
BSA	AAI	1.89 × 10^6^	1.18	0.9912	0.0046
	AAII	5.00 × 10^6^	1.23	0.9933	0.0036
