# Supplementary material for: Mass Spectrometric and Spectrofluorometric Studies of the Interaction of Aristolochic Acids with Proteins
Source: Sci Rep. 2015 Oct 16;5:15192. doi: 10.1038/srep15192 (PMC4608009; doi:10.1038/srep15192)
Supplement: Supplementary Information [file srep15192-s1.doc]

**Supporting Information**

**Mass Spectrometric and Spectrofluorometric Studies of the Interaction of Aristolochic Acids with Proteins**

Weiwei Li, Qin Hu, and Wan Chan *

Department of Chemistry, The Hong Kong University of Science and Technology, Clear Water Bay, Kowloon, Hong Kong

* Correspondence author. Phone: (852) 2358-7370; Fax: (852) 2358-1594; E-mail: [chanwan@ust.hk](mailto:chanwan@ust.hk).


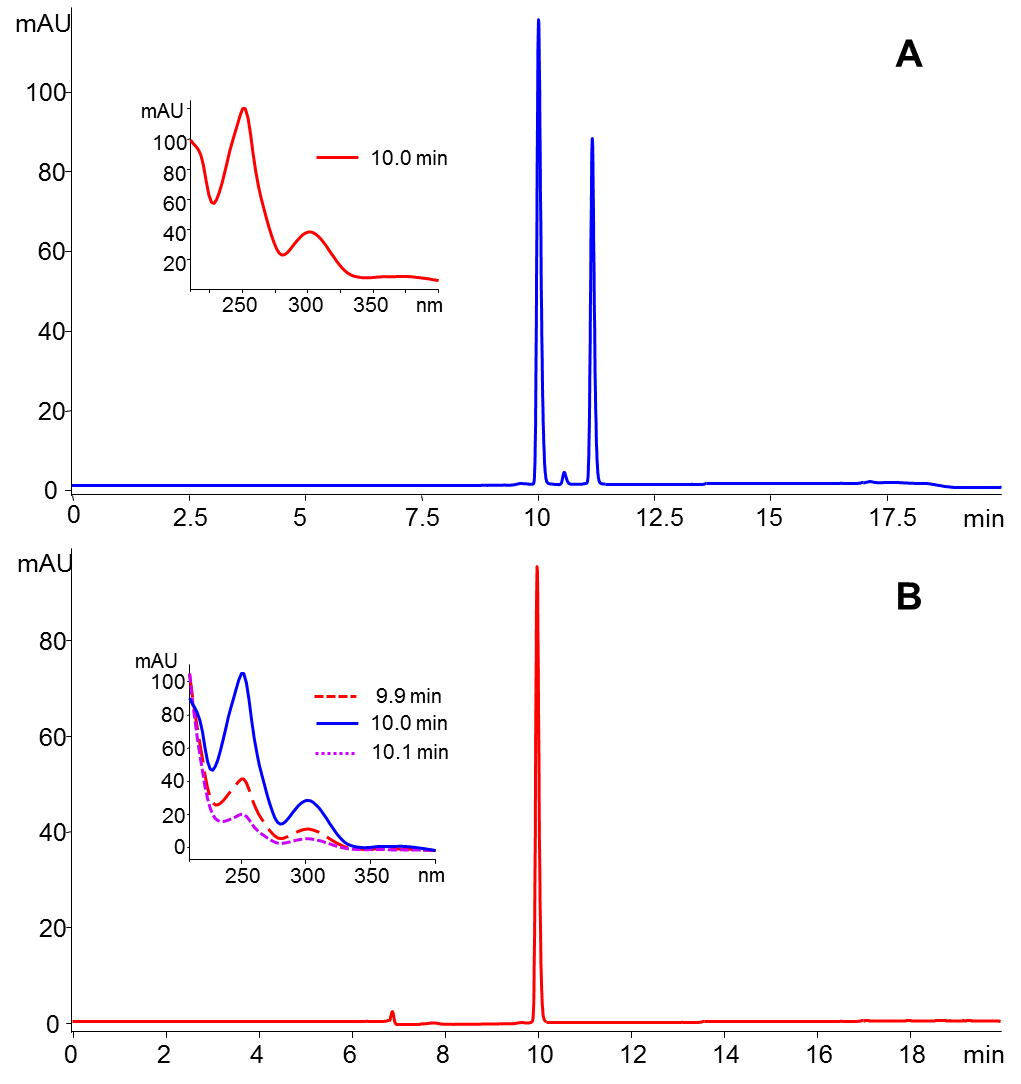


**Figure S1.** HPLC analyses of (A) authentic standards of AAI and AAII mixture; and (B) AAII isolated from *Aristolochiae Cinnabarina*. AAI and AAII were eluted at retention time of 11.1 and 10.0 min, respectively. Shown in the insets are the UV absorbance spectra of AAII.
